# Examining capabilities, opportunities, and motivations for healthy eating behaviors in Latin American restaurants: a quantitative application of the COM-B model to inform future interventions

**DOI:** 10.1186/s40795-023-00712-1

**Published:** 2023-03-27

**Authors:** Melissa Fuster, Maria P. Santos, Emily Dimond, Terry T. K. Huang, Margaret A. Handley

**Affiliations:** 1 Department of Social, Behavioral, and Population Sciences, School of Public Health and Tropical Medicine, Tulane University, New Orleans, LA USA; 2 Department of Epidemiology, School of Public Health and Tropical Medicine, Tulane University, New Orleans, LA USA; 3 Center for Systems and Community Design and NYU-CUNY Prevention Research Center, City University of New York Graduate School of Public Health and Health Policy, New York, NY USA; 4 Partnership for Research in Implementation Science for Equity (PRIDE) Center and Department of Epidemiology and Biostatistics, School of Medicine, University of California, San Francisco, CA USA

**Keywords:** Restaurants, Foods away from home, Eating behaviors, Theoretical Domains Framework, COM-B Model, Latin/Hispanic

## Abstract

**Background:**

Eating foods away from home has been associated with poor diet quality and adverse health outcomes. Research is needed to examine barriers and facilitators to making healthier eating choices in restaurant settings. We operationalized the Capability, Opportunity, and Motivation for Behavior Model (COM-B Model) to conduct a behavioral diagnosis for healthy eating behaviors at Latin American restaurants (LARs), an understudied yet increasingly important food environment with the potential to positively influence diets.

**Methods:**

We conducted an online survey with adults in the United States that reported eating food from LARs at least once a month (*n* = 509) recruited via an online market research panel to examine capabilities – physical (e.g., skills) and psychological (e.g., knowledge), opportunities – social (e.g., norms) and physical (e.g., environmental), and motivations – reflective (e.g., self-conscious intentions) and automatic (e.g., emotions) associated with healthier choices at LARs. In a survey focused on LAR-associated behaviors, each COM-B domain was scored between 1–5, with scores ≥ 4 denoted as having high capability, opportunity, and motivation to eat healthfully at LARs (potential range of total score = 6–35). Regression analysis was used to examine the association between COM-B scores (total and by domain) and select demographic characteristics (age, gender, race, Latin heritage, income, education, marital status, and Latin majority state of residency).

**Results:**

More than half of the participants (57.1%) were classified as having high physical capability, followed by psychological capability (43.9%) in the LAR environment. The proportions of participants with either high motivation or high opportunity were low, ranging from 37.3% (reflective motivation) to physical opportunity (15.6%). The overall mean COM-B total score was 19.8 ± 3.0. Higher total COM-B scores were associated with younger age, self-identifying as white, having Latin heritage, and having higher income (*p* < 0.05).

**Conclusions:**

This study expands the application of the COM-B framework using quantitative inquiry to evaluate levels of capability, motivation, and opportunity for healthy eating in LAR settings and initial demographic associations with determinants for healthy eating in these settings. This work can aid in tailoring interventions and developing evaluation tools for LAR-related healthy eating interventions.

## Background

While public health policies and interventions have been increasingly focusing on restaurants as a sector for intervention, these efforts often fail to include establishments serving minority communities, exacerbating existing diet-related health inequities. According to the National Restaurant Association, 80% of consumers eat at a restaurant serving ethnic cuisine at least once a month [1]. Within these, there are over 120,000 Latin American restaurants (LARs) in the United States (US), most of which are independently owned. Mexican restaurants alone make up 8% of all US restaurants [2, 3]. Yet, despite their importance, LARs (along with other ethnic restaurants) remain an understudied sector, where factors associated with consumer food choices in these settings are not well understood. This gap is important, given the increased consumption of foods away from home and its association with poor dietary outcomes [4]. Diet-related non-communicable diseases, such as heart disease and diabetes, are among the leading causes of death globally. In the U.S., minority populations, including Latins/Hispanics, are disproportionately affected by these conditions.

Research examining consumer choices in restaurants show that several customer attributes are associated with nutrition considerations when choosing a meal, including knowledge of health issues, weight concerns, gender, age, and marital status [5]. This research has not taken place in ethnic eateries, where there may be additional factors influencing food choices and availability, given the importance of culture and notions of authenticity [6, 7]. Theory-driven research is needed to provide evidence for future intervention and policies that can best address the added level of complexity when seeking to promote behavior change related to establishments serving ethnic cuisines. This research need presents an opportunity for the application of implementation theories and frameworks to address a complex, community-based evidence-to-practice gap.

Determinant frameworks, such as the Capability, Opportunity, and Motivation for Behavior (COM-B) Model can help identify key drivers of dietary behaviors for informing intervention design, as part of the Behavior Change Wheel, an intervention development framework that brings together theory-based tools to understand and change behaviors [8]. The COM-B model synthetizes theoretical frameworks to understand a given behavior within interacting factors – capability, opportunity, and motivation. Capability is addressed as physical (i.e., whether the individual has the needed skills to engage in the desired behavior) and psychological (i.e., an individual’s comprehension and knowledge). Opportunity encompasses the external factors influencing the behavior, including physical (i.e., aspects of the built and food environments that influence behavior) and social opportunity (i.e., cultural and social norms that influence the behavior). Lastly, motivation encompasses the internal processes that direct behavior, including reflective (i.e., evaluations and plans) and automatic (emotions) motivation [8]. The COM-B Model has been used to understand a wide range of behaviors, including eating behaviors [8–10]. However, the research has been mostly qualitative. Willmott and colleagues present an exception, operationalizing COM-B to examine general healthy eating behaviors among youth, demonstrating the predictive utility of COM-B for general healthy eating behaviors [11]. In this study, we built on these previous applications to [1] develop a short COM-B-based questionnaire to assess determinants of making healthier eating choices in LARs, and [2] examine the demographic factors associated with COM-B.

## Methods

### Survey development

We developed a cross-sectional survey that operationalized the six COM-B domains into a short scale. The items were developed building on previous research that operationalized the COM-B [11, 12]. The resulting scale was composed of 14 items, encompassing thirteen 5-point Likert-scale questions and one open-ended question. The 5-point Likert-scale questions asked respondents to rate the level of agreement with a given statement and were scored according to how the response supported the given COM-B domain. The psychological capability domain was examined using an open-ended question to assess knowledge about healthful dishes in Latin American cuisines, designed to accommodate the potential variety in responses, given the diversity within the Latin American community. The scale was pre-tested among a small number of respondents (*n* = 5) who met the survey inclusion criteria and revised for language clarification.

### Data collection

Data were collected using a web survey between April and July 2021 distributed via social media and through Centiment, an online market research panel. The survey was available in English and Spanish. Participants were screened for eligibility prior to the full survey application. Inclusion criteria for the study were: being an adult (18 years of age or older), living in the 50 US states or Puerto Rico, and eating at or ordering from restaurants specializing in Latin American cuisines at least once a month.

The survey took about 10–12 min to complete. In addition to the COM-B questions, we collected demographic information as follows: Income was assessed as individual annual income, using eight categories in the survey, ranging from $0 to $150,000 or greater. Education was assessed as the highest level attained (less than high school, high school/GED, some college, bachelor's degree, post-graduate education). Gender was assessed as male, female, transgender woman, transgender man, and non-binary. Latin/Hispanic background was self-reported through the question, “Do you have a Latin/Hispanic heritage?” (Yes/No), and we included a question to collect specific heritage information. Race was collected using distinct categories (White, Black/African American, Asian, American Indian/Alaska Native, Pacific Islander, Mixed/Multi-racial) and a write-in option. Age was collected as a continuous variable. We also collected information about employment status, marital status, and place of residence.

### Data analysis

The Likert-scale responses were scored according to level of support for COM-B domains, using a scale of 1–5. The qualitative responses for psychological capability were coded by two raters independently, initially using a scale of 0–2. Response that clearly showed a lack of knowledge regarding potentially healthful Latin foods were scored as “0”, including those who responded “I don’t know,” or who listed unhealthful options (e.g., fried foods). Responses that provided a potentially healthy Latin food but lacked specificity or clarity for why they were healthy were scored as "1". Examples included “chicken” and “a vegetarian dish.” Responses that clearly showcased knowledge of healthy Latin foods were scored as "2". Common examples included a specific food like ceviche (a seafood-based dish) or a healthier preparations of a usual dish like “baked chicken” (instead of fried). The raters were trained in qualitative coding, and they had nutrition training and knowledge of Latin American cuisines. Independent scores were reconciled by the raters, in collaboration with the lead author and study principal investigator, where the team discussed the responses in disagreement until a common score was reached. Responses were recoded into a 1–5 scale, to conform with the rest of the scale items.

The six individual COM-B domain scores were calculated as the average of responses received within the individual items related to the domain, to avoid giving more weight to domains where more items were incorporated. The total COM-B score was calculated by summing up the resulting individual six scores, with a potential range of 6–30, where higher scores denoted greater capability, opportunity, and motivation to make healthier choices at LARs. Confirmatory factor analysis was used to validate the COM-B in our sample, using variance–covariance matrix with maximum likelihood estimation and varimax rotation.

For descriptive, exploratory analysis, domain scores were transformed into a categorical variable to denote high scores, using the cut-off of 4 or higher. However, total and domain scores were analyzed as continuous variables given the resulting distribution of the exploratory categorical variables. Mean and standard deviations were computed for each item in the COM-B scale, as well as the total COM-B score and domain scores. Scores were normally distributed. We carried out descriptive statistical analysis as part of the sample description and preliminary bivariate analysis to examine the association between demographic characteristics and COM-B domains and total scores. We then carried out multilinear regression analysis to examine the association of select socio-demographic characteristics with the COM-B scores, namely, age, race, Latin heritage, gender, marital status, educational attainment, income, and place of residence. Age was analyzed as a continuous variable. Due to data distribution, race was collapsed as a binary variable, denoting respondents identifying as white, compared to those selecting other categories (non-white). Latin heritage was assessed as binary (yes/no), with specific heritage reported only as part of sample description, due to small cell counts for more specific analysis. Gender was analyzed as male and female, due to the small sample (*n* = 3) self-identifying within nonbinary categories, which were set to missing. The education variable was analyzed as a binary variable comparing respondents with less than a bachelor’s degree to those with a bachelor’s degree or higher. Income was examined as Low (< $25,000), Middle ($25,000-$74,999) and High (> $75,000) based on the 2020 US median personal income and the Pew Research Center income classification method [13, 14]. Marital status was analyzed as a binary variable (married/living with someone compared to single/divorced/separated) (no widowed person in sample). Place of residence was collapsed into a binary variable, denoting if the state had an above average percentage of Latin/Hispanic population (12% according to 2010 Census data) [15]. The analysis was conducted using SAS 9.4 and STATA 11.2. Significance was set at *p* < 0.05, but we also noted marginally significant associations at *p* < 0.10, given the exploratory nature of the analysis.

## Results

### Sample characteristics

More than half of the survey sample was comprised of those who are white, female, married, and employed (Table 1). A larger percentage (46%) was classified as middle income than lower (23%). Regarding educational attainment, there was a close to even split between respondents with a bachelor’s degree or higher, and those with some college or below. More than half of the sample reported residing in a US state/location with above average percentage of Latin population. The top locations were California (11.3%), Texas (10.8%), New York (9.3%), and Florida (9.0%) – the states with the highest population of Latinos in the US. Close to half of the sample reported having Latin heritage. Regarding racial self-identification, most with Latin heritage identified as white (41.5%) or mixed/other (36.2%); only a few identified as Black (6.3%).

**Table 1:** Sample Description (*n = *509, except where noted)

Sample Characteristics	n (%) or mean ± SD
Race	
White	341 (67.3)
Black	32 (6.3)
American Indian / Alaska Native	6 (1.2)
Asian	17 (3.4)
Native Hawaiian / Pacific Islander	3 (0.6)
Multiracial	84 (16.6)
Latin/Hispanic heritage (% yes)	210 (41.3)
Age	47.1 ± 18.3
Gender	
Woman	273 (53.5)
Man	230 (45.3)
Gender minority/non-binary	3 (0.6)
Marital status	
Married/living with someone	267 (53)
Single	155 (30.8)
Widowed/Divorced/separated	76 (15.1)
Income (*n* = 468)	
Low (< $25,000)	110 (23.4)
Middle ($25,000–74,9999)	217 (46.4)
High (≥ $75,000)	141 (30.1)
Education (*n* = 492)	
No post-secondary education	91 (18.4)
Some college	138 (27.9)
Bachelor’s	160 (32.3)
Graduate degree	106 (21.4)
Employment	
Employed	298 (62.2)
Unemployed	50 (10.4)
Retired	131 (27.4)
% Living in state with above average Latin/Hispanic population	271 (53.6)

### Determinants of making healthy choices in LARs: COM-B assessment

Individual COM-B item mean scores ranged from 2.86 to 3.80, with the highest score found for being willing to try new healthier foods when eating at LARs (Motivation-Reflective). In CFA, using three factors, item loading ranged from 0.41 to 0.96, with all items above 0.4 (Table 2). The resulting Goodness of Fit Index and Adjusted Goodness of Fit Index were 0.98 and 0.92, respectively.

**Table 2:** COM-B scale item mean scores and factor loading, with resulting mean domain scores distributions (*n* = 504)

COM-B Domain	Scale Item^1^	Item Score (mean ± SD)	Factor Loading in CFA	Domain Score (mean ± SD)	% with High Domain Scores^2^ n (%)
Capability-Psychological (Knowledge)^3^	When you think of healthy, or “good for you” dishes in Latin cuisines, what comes to mind?	3.02 ± 1.92	0.41	3.02 ± 1.92	224 (46.5%)
Capability-Physical (Skill)	It is easy to identify healthy options when visiting Latin restaurants	3.57 ± 0.92	0.52	3.57 ± 0.92	289 (57.1%)
Opportunity -Social (Social influences)	Most of my friends and family tend to order healthy options when visiting Latin restaurants	3.56 (0.87)	0.55	3.08 ± 0.64	52 (10.3%)
	I expect to find healthy options when visiting Latin restaurants	3.14 (0.98)	0.63		
	Authentic Latin dishes are not really healthy.^a^	2.94 (1.03)	0.57		
Opportunity-Physical (Environmental constraints)	Latin restaurants tend to offer a good variety of healthy and appealing choices	3.45 (0.95)	0.96	3.25 ± 0.62	79 (15.6%)
	Healthier choices in Latin restaurants tend to be more expensive than other, less healthy choices. ^a^	2.86 (1.06)	0.42		
	Latin restaurants tend to serve too much food. ^a^	3.45 (0.95)	0.96		
Motivation-Reflective (Self-efficacy, plans)	I am willing to try new healthier foods when eating at Latin restaurants	3.80 (0.96)	0.67	3.65 ± 0.62	189 (37.3%)
	It is OK to indulge in foods that may not be healthy when eating at Latin restaurants	3.66 (0.94)	0.41		
	Ordering healthy choices at Latin restaurants will have a big positive impact in my overall health	3.62 (0.98)	0.63		
	I want to eat healthy dishes at Latin restaurants	3.53 (0.92)	0.71		
Motivation-Automatic (Emotions, reinforcements)	Eating healthier choices at Latin restaurants makes me like feel I am restricting myself. ^a^	3.01 (1.09)	0.42	3.29 ± 0.73	122 (24.1%)
	I tend to feel good physically when I select lighter meals in Latin restaurants	3.58 (0.93)	0.65		

^1^With the exception of psychological capability, all items scored based on responses to Likert-scale responses (1 = Strongly disagree; 2 = Disagree; 3 = Neither agree nor disagree; 4 = Agree; 5 = Strongly agree). Items marked with (^a^) denote reverse scoring, where disagreement was scored higher;

^2^Scores ≥ 4;

^3^Smaller sample size for psychological capability, *n* = 482

The mean COM-B domain scores ranged from 3.02 (Capability-Psychological) to 3.65 (Motivation-Reflective) (Table 2). Within a possible range of 6–30, the total mean COM-B score was 19.8 ± 3.0, ranging from 11.6 to 30.

When score distributions were examined as the proportion within the sample scoring high (≥ 4) in each COM-B domain, the domains with the highest percentage of high scores were those related to capability, where more than half or close to half of the sample were classified as having the physical and psychological capability, respectively, to select healthier choices at LARs (Table 2). The prevalence of high scores was much lower for the motivation and opportunity domains. While only approximately one-third of respondents had high motivation to select healthier choices at LARs, even fewer were classified as having the social and physical opportunities needed to engage in the desired behavior (Table 2).

**Fig. 1:**
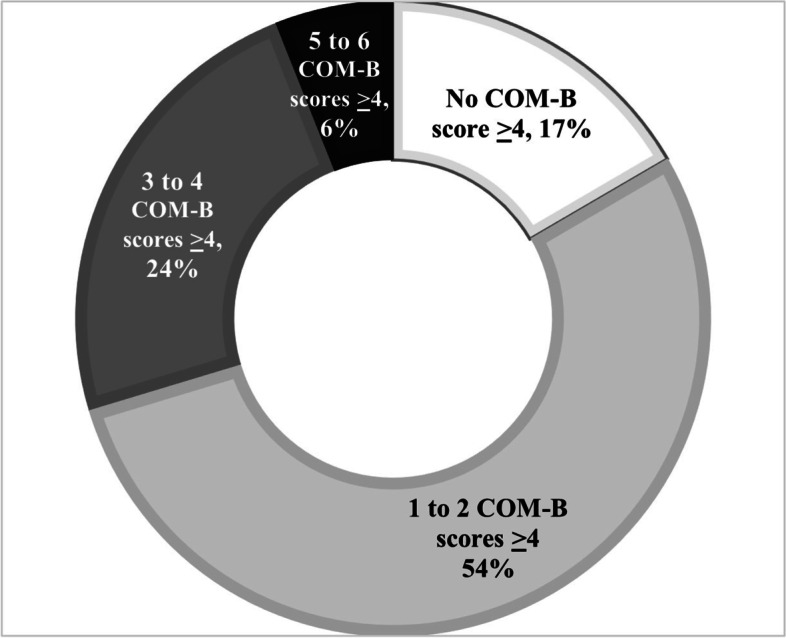
Distribution of total COM-B domains with high (≥ 4) scores, *n* = 489

On average, respondents had a total of 1.9 ± 1.4 domains with high scores. Very few respondents had high scores across all or most of the six domains; more than half presented only one to two high scoring domains (Fig. 1).

**Fig. 2:**
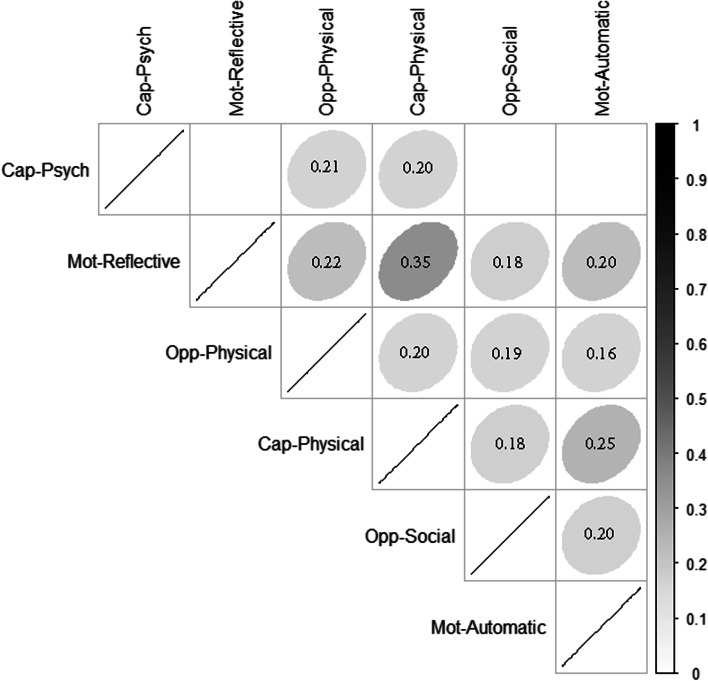
Proportion of participants with overlapping high scores by domain dyads

We found overlaps among participants scoring high in specific domains. Figure 2 denotes the proportion of participants with overlapping high scoring domains, darker gray color denotes a higher proportion of participants with high scores in both corresponding domains in the y and x axis. Physical capacity and reflective motivation had the highest proportion of overlapping high scores (35% of participants), followed by automatic motivation and physical capacity (25% of participants) and reflective motivation and physical opportunity (22% of participants; Fig. 2).

### Sociodemographic factors associated with higher COM-B scores

Higher total COM-B scores were associated with younger age, being white, having Latin heritage, being a woman, higher income, and living in a state with above average percentage of Latin/Hispanic population (Table 3). Associations also across domain scores. Age and race were significantly associated across 5 of the 6 domains (except for psychological capability, which was significantly associated with gender—higher among women—and higher education). For age, younger customers had higher scores, except for physical opportunity, where increasing age was associated with increased score. Latin heritage was positively associated with higher social opportunity and automatic motivation. Aside from higher psychological capability, being a woman was associated with higher reflective motivation, but marginally lower automatic motivation (*p* < 0.10). Income was marginally associated with the automatic motivation domain, where medium income compared to low income was associated with lower scores (*p* < 0.10). Lastly, living in a location with above average percentage of Latin/Hispanic population was only associated with higher social opportunity. The total COM-B score showed the highest number of significantly associated demographic factors. Across the six domains, automatic motivation showed the most associations with demographic factors (5 out of 6), followed by social opportunity, with 4 out of 6 (Table 3).

**Table 3:** Multilinear Regression results examining COM-B scores (total and by domain) against sociodemographic factors (*n* = 429)

	COM-B	Capability-Psychological	Capability-Physical	Opportunity-Physical	Opportunity-Social	Motivation-Reflective	Motivation-Automatic
	β (se)	β (se)	β (se)	β (se)	β (se)	β (se)	β (se)
Age	-0.026 (0.009)**	0.009 (0.006)	-0.008 (0.003)**	0.005 (0.002)*	-0.008 (0.002)***	-.006 (0.002)**	-0.007 (0.002)**
Race							
Non-White	REF	REF	REF	REF	REF	REF	REF
White	1.24 (0.37)**	0.007 (0.242)	0.313 (0.112)**	0.124 (0.075)^	0.253 (0.077)**	0.222(0.077) **	0.387 (0.091)***
Latin Heritage							
No	REF	REF	REF	REF	REF	REF	REF
Yes	0.977 (0.342)**	0.282 (0.224)	0.151 (0.105)	0.115 (0.070)	0.227 (0.072)**	0.052 (0.071)	0.159 (0.084)^
Gender							
Woman	REF	REF	REF	REF	REF	REF	REF
Man	-0.517 (0.288)^	-0.412 (0.188)*	-0.096 (0.089)	-0.001 (0.059)	0.042 (0.061)	-0.120 (0.060)*	0.120 (0.071)^
Marital Status							
Single/divorced/ separated	REF	REF	REF	REF	REF	REF	REF
Married/Living with someone	0.206 (0.305)	0.123(0.200)	-0.024 (0.094)	0.012 (0.060)	0.015 (0.065)	0.0314 (0.064)	0.072 (0.076)
Educational Attainment							
Some college or less	REF	REF	REF	REF	REF	REF	REF
Bachelor’s or higher	0.317 (0.294)	0.438 (0.193)*	-0.052 (0.091)	-0.028 (0.060)	-0.042 (0.062)	0.045 (0.062)	-0.025 (0.073)
Individual Income							
Low (< $25,000)	REF	REF	REF	REF	REF	REF	REF
Medium ($25,000-$74,999)	-0.136 (0.369)	0.021(0.242)	-0.026 (0.114)	-0.063 (0.075)	-0.002 (0.078)	0.046 (0.078)	-0.162 (0.092)^
High ($75,000 +)	0.918 (0.438)*	0.437 (0.288)	0.216 (0.136)	0.081 (0.090)	0.11 (0.093)	0.105 (0.093)	0.054 (0.109)
Living in location with above average Latin/Hispanic population							
No	REF	REF	REF	REF	REF	REF	REF
Yes	0.592 (0.283)*	0.168 (0.186)	0.024 (0.087)	0.020 (0.058)	0.104 (0.060)^	0.120 (0.059)	0.049 (0.070)

Levels of significance: ^*p* < 0.10, ^*^p < 0.05, ^**^p < 0.01, ^***^p < 0.001

## Discussion

This study applied a theoretical framework to examine capabilities, opportunities, and motivation associated with healthier eating behaviors in LARs, an understudied, yet increasingly important community food source. The application of the COM-B model allows a systematic exploration of behavior, in this case, a range of behaviors associated with selecting healthier foods at LARs. The examination revealed that, while interventions may be needed across all areas of the COM-B, there is a pressing need to address physical and social opportunities, as the domains found most lacking among the respondents, to make a strong impact on these LAR environment healthy eating behaviors. Physical opportunity is having an environment that affords the time, resources, location, and other physical affordances associated with the desired behavior. The association between food environments and healthier food consumption has been documented [16], noting linkages between healthful environments and cardiovascular disease risks [17]. In this study, we focused on the availability of appealing healthy options that are also affordable and in adequate portions. While food environment research to date underscores the need to increase availability and accessibility of healthy options, this paper illustrates the importance of norms and notions associated with foods. These factors are captured under the social opportunity domain, referring to interpersonal influences and sociocultural norms that enable the desired behavior [8]. Social norms can influence food consumption via peer influence and also by changing perceptions of certain foods, with the potential for making healthier options more appealing [18, 19]. In restaurant setting, social perceptions concerning establishment types have the potential to influence food choices, regardless of actual food availability. For instance, notions of fast food restaurants as places for junk food consumption have been associated as a barrier for healthy eating at these establishments [20]. Moreover, research has also started to examine the role of hedonic descriptions to motivate healthier food consumption in restaurant menus [21].

The application of the COM-B as part of the larger, related intervention design framework – the Behavior Change Wheel Model – allows for the identification of potential intervention functions to promote desired changes. Physical and social opportunity can be enhanced via environmental restructuring, restriction, and enablement. These changes imply interventions beyond individual-level strategies and the importance of changing consumer nutrition environments in LARs, not only by increasing the variety of healthier food options, but also by enabling healthier choices through, for example, menus highlights. Emerging evidence from restaurant intervention studies suggests that restaurants that increase the availability of healthier options and enable healthier choices can result from increased consumption [cite/expand]. These changes can also boost social opportunity, showcasing healthy options through innovative approaches that not only underscore the health benefit of these choices but also showcases these healthier options as traditional and palatable. While most interventions, to date, have focused on augmenting physical opportunity, more attention is needed to address social norms concerning healthier foods. These approaches can help increase interest in these offerings, while also normalizing these healthier options in the community, which, in turn, can help foster changes in social and cultural norms about which foods customers should expect to find in these establishments.

Our study’s operationalization of the COM-B model facilitated a statistical analysis to examine demographic characteristics associated with having the capabilities, opportunities, and motivations necessary to engage in healthier eating behaviors at LARs. Age and race were consistently associated across all domains, except for psychological capability. The results suggest that younger customers may have more social opportunity, motivation, and skills (physical capability) to engage in healthier eating behaviors at LARs. This corroborates previous research showing greater interest in healthier eating among younger generations [22]. On the other hand, older age was associated with physical opportunity, which may point to age-related differences in perceived physical and economic access to healthy choices in LARs.

The significant association with race shows the potential racial inequities in facilitators for healthier choices in LARs. While most studies tend to examine race and ethnicity, incorporating “Hispanic/Latino” as a category, our study examined race and Latin heritage separately, allowing for a more nuanced exploration of these demographic characteristics. Our results suggest that self-identifying as white was associated with more COM-B domains, compared with having Latin heritage. These findings showcase the importance of examining racial differences among Latin/Hispanic communities, an important research need documented in past research [23].

Gender was associated with higher psychological capability and motivation. This coincides with research that examines gender difference in interest in healthy eating behaviors. In restaurant settings, women tend to worry more about the caloric content of foods, compared to men [24]. Healthier foods, like vegetables, fruit, and fish, are typically associated with femininity, and women are usually more aware of the health-diet relationship than men [25]. Research examining food purchasing behaviors by gender is scarce, and it suggests that men tend to consume more foods away from home, compared with women, but no significant differences in diet quality were found [26]. More research is still needed to elucidate gender differences, including more studies that focus on men and gender minorities.

Variables associated with socioeconomic status (income and education) did not show associations across most COM-B domains. Income was only significantly and positively associated with total COM-B score. Higher education was only significantly associated with greater psychological capability (knowledge). Hence, while socioeconomic factors have been documented as important factors associated with diet quality [27, 28], economic access appears to be only one part of the story for food choices in restaurant settings. However, more research is needed to elucidate differences in food consumption in restaurants by socioeconomic characteristics, building on emerging work examining dietary intakes by food source [29].

Lastly, we found that residence in a state with an above average percentage of Latin population, was significant only for the total COM-B score and social opportunity. We expected to find that a higher proportion of Latin/Hispanic population may result in higher exposure to LARs and Latin food in markets [30], and that this exposure may, in turn, influence aspects of the COM-B, such as through increased knowledge of Latin foods or greater social opportunity, for example. Our results suggest some association, but more research is needed. Our research was limited, as we assessed Latin/Hispanic population at the state level, which fails to capture neighborhood-level concentrations – an approach that can be explored in future research. While research documents the potential benefits of ethnic enclaves through greater availability of relevant cultural institutions, like restaurants, more work is needed to assess how such restaurants may be perceived by others in the area. Recent work examining how immigrant-run food establishments are perceived show different results, where in some cultural diversity in food store availability is viewed as a positive [31], whereas in another work these establishments are perceived to be of lower quality [32]. However, more research is still needed to examine potential associations between the availability of ethnic restaurants and food choices.

The present assessment applied the COM-B in a complex, community-based setting, moving this area of research beyond mostly clinical settings and qualitative approaches in past studies. However, there are some limitations in the study. First, our measures were based on self-reports which might be biased by social desirability. Second, to keep the scale short, some aspects of the COM-B, particularly the capability measures, were assessed with only one item. In addition, knowledge was assessed through an open-ended question because we wanted to capture consumers’ interaction with a rich diversity of offerings in complex LAR settings. Future research is warranted to further validate and refine the survey tool. Third, while this study enhanced our understanding of demographic group differences in COM-B, future research should extend the research to objective measures of restaurant purchases and consumption as well as the consideration of other factors that influence eating behaviors at restaurants, such as prices, type of eating occasion, presence of others, and type of restaurant. Finally, our findings are not generalizable to the larger population. However, the distinct associations found in this initial assessment suggest that this may be a promising assessment for aiding in targeting interventions.

## Conclusion

Foods away from home are an increasingly important food source, including from LARs. More research is needed to understand potential enablers and barriers for healthy eating behaviors in these settings and to develop effective multi-level interventions with the potential to positively influence dietary health. The examination of COM-B in relation to LAR-associated behaviors provides an advancement in this area of research, while also extending the application of the underlying theoretical framework. The survey developed in this research can be adapted and expanded for application in intervention design and evaluation studies, as a feasible tool that can be applied as part of other data collection efforts. Greater understanding of the role of ethnic cuisines and how consumers interact with these restaurants is critical to addressing diet-related chronic diseases and reducing health disparities.

## Data Availability

De-identified data and materials supporting findings are available upon reasonable request from the corresponding author.

## Abbreviations

COM-B Model: Capabilities, Opportunities, and Motivation for Behavior Model
LARs: Latin American restaurants
US: United States
